# Disease spectrum, prevalence, genetic characteristics of inborn errors of metabolism in 21,840 hospitalized infants in Chongqing, China, 2017-2022

**DOI:** 10.3389/fgene.2024.1395988

**Published:** 2024-05-28

**Authors:** Dongjuan Wang, Juan Zhang, Rui Yang, Dayong Zhang, Ming Wang, Chaowen Yu, Jingli Yang, Wenxia Huang, Shan Liu, Shi Tang, Xiaoyan He

**Affiliations:** ^1^ Center for Clinical Molecular Medicine, National Clinical Research Center for Child Health and Disorders, Ministry of Education Key Laboratory of Child Development and Disorders, China International Science and Technology Cooperation Base of Child Development and Critical Disorders, Chongqing Key Laboratory of Pediatrics, Children’s Hospital of Chongqing Medical University, Chongqing, China; ^2^ Department of Neonatology, National Clinical Research Center for Child Health and Disorders, Ministry of Education Key Laboratory of Child Development and Disorders, Chongqing Key Laboratory of Pediatrics, Children’s Hospital of Chongqing Medical University, Chongqing, China

**Keywords:** inborn errors of metabolism, newborn screening, disease spectrum, genetic characteristics, tandem mass spectrometry

## Abstract

Inborn errors of metabolism (IEMs) are uncommon. Although some studies have explored the distribution and characteristics of IEMs in newborns, the impact of these disorders on hospitalized newborns remains unclear. In this study, we gathered data from 21,840 newborn patients admitted for various medical conditions at the Children’s Hospital of Chongqing Medical University from January 2017 and December 2022. Liquid chromatography-tandem mass spectrometry (LC-MS/MS), gas chromatography-mass spectrometry (GC-MS/MS), and genetic analysis were used to elucidate the disease spectrum, incidence rate, and genetic characteristics of IEMs in hospitalized newborns. The results revealed that the incidence of IEMs in hospitalized newborns was 1/377 (58/21,840), with a higher incidence in full-term infants (1/428) than in premature infants (1/3,120). Among the diagnosed genetic metabolic diseases, organic acid metabolism disorders (1/662), amino acid metabolism disorders (1/950), and fatty acid oxidation disorders (1/10,920) were the most prevalent. Methylmalonic acidemia (MMA), especially the isolated form, emerged as the most common IEM, while neonatal intrahepatic cholestasis caused by citrin deficiency (NICCD) and ornithine transcarbamylase deficiency (OTCD) were prevalent in premature infants. Of the 58 confirmed cases of IEMs, 72 variants were identified, of which 31.94% (23/72) had not been reported previously. This study contributes to understanding the incidence and clinical features of IEMs in hospitalized newborns, offering more efficient strategies for screening and diagnosing these disorders.

## 1 Introduction

Inborn errors of metabolism (IEMs) constitute a group of disorders caused by defective enzymes, cofactors, or transporters in the metabolic pathway, leading to the abnormal accumulation of metabolites and deficiencies of essential substances. The disease profile encompasses amino acid metabolism disorders (AAMD), organic acid metabolism disorders (OAMD), and fatty acid oxidation disorders (FAOD). Although the prevalence of individual conditions was low, the overall incidence rate of IEMs ranged from 1/2,500 (Tebani et al., 2016) to 1/3,000 (Patial et al., 2022). With the availability of new biochemical phenotypes techniques, more and more IEMs were being identified, and the cumulative incidence of IEMs has steadily increased to more than 1/800 (Mak et al., 2013). IEMs can manifest across in all age groups but were most commonly observed in early life. The clinical manifestations of IEMs varied widely, ranging from mild symptoms to severe and life-threatening conditions. Early clinical signs such as feeding difficulties, seizures and lethargy, were often atypical, posing challenges in diagnosis during the initial stages, which in turn resulted in disability, deformity and even death (Saudubray and Garcia-Cazorla, 2018). The same disease tended to present with significant phenotypic variability due to individual differences, and different diseases also exhibited similar phenotypes, making diagnosis challenging. Considering that pre-symptomatic diagnosis was crucial for timely intervention, especially for treatable IEMs, early screening and diagnosis have become important strategies for reducing mortality and morbidity rates in children with IEMs (Cossu et al., 2023).

Tandem mass spectrometry (MS/MS), including liquid chromatography-tandem mass spectrometry (LC-MS/MS) and gas chromatography-mass spectrometry (GC-MS/MS), is the most universal technology for the screening and diagnosis of IEMs. LC-MS/MS was first used in the newborn screening in 1990. Currently, it served to screen for organic acid, amino acid and fatty acid metabolic diseases by detecting various amino acids and acylcarnitines in the dried blood spot (DBS), with advantages of easy operation, high throughput and rapid detection speed (Patial et al., 2022). However, some IEMs lack specific biochemical markers in blood, particularly in the early stages of the disease, and cannot be recognized and diagnosed using LC-MS/MS alone. GC-MS/MS, originally used in 1966 to diagnose isovaleric academia, is a valuable complementary tool (Holmes et al., 1966). GC-MS/MS primarily assessed dozens of organic acids in urine to differentiate IEMs, providing additional support to diagnose IEMs. To date, MS/MS has been extensively used for the diagnosis of IEMs in high-risk infants and children in the United States of America, most European countries, Asia, and some Arab nations (Lim et al., 2014; Selim et al., 2014). In China, LC-MS/MS and GC-MS/MS were gradually integrated into the screening and diagnosis of IEMs at the beginning of the 21st century (Han et al., 2008). This reflected the global recognition of MS/MS as an indispensable technology for IEM screening and diagnosis.

Although MS/MS has powerful utility in the screening and diagnosis of IEMs, gene sequencing is still necessary for certain conditions characterized by common abnormal metabolites or the absence of specific metabolites. As a follow-up test for infants with abnormal MS/MS screens, gene tests, including targeting of hundreds of IEMs-associated genes (Yang et al., 2023), whole exome sequencing (WES) (Aashish et al., 2020), and rapid whole-genome sequencing (rWGS) (Kingsmore et al., 2022), contributed to early diagnosis and treatment of IEMs. As a tool for IEMs screening, WES demonstrated an overall sensitivity of 88% and specificity of 98.4%, compared to 99.0% and 99.8%, respectively, for MS/MS (Martín-Rivada et al., 2022). Despite its slightly inferior performance to that of MS/MS, sequence-based detection of IEMs could reduce false-positive results of MS/MS, facilitate timely case resolution, and, in some instances, even suggest a more appropriate or specific diagnosis than that initially obtained through MS/MS alone.

With the rapid development of MS/MS and gene sequencing technology, as well as the advancement of medical genetics research on IEMs, an increasing number of reports on the distribution and characteristics of IEMs were emerging. The incidence of IEMs varied in different countries and populations worldwide and was approximately 1/2,670 in the Madrid region (Martín-Rivada et al., 2022), 1/8,557 in Japan (Shibata et al., 2018) and 1/3,367 in the United States (Frazier et al., 2006). In China, the incidence of IEMs ranged from 1/1,683 to 1/8,304 (Guo et al., 2018) in newborns, while some studies on high-risk children with IEMs have reported even higher rates of incidence, such as 1:51 (based on 6,210 cases) (Sun et al., 2011), or 1:53 (based on 16,075 cases) (Jiang et al., 2015). The incidence rates in different populations should also be considered. Most IEMs manifest in the neonatal period, making it necessary to evaluate the impact of IEMs on the disease status of newborns requiring hospitalization, especially those admitted to the neonatal intensive care unit (NICU) due to severe symptoms (El-Hattab, 2015). Although a few studies on the distribution of IEMs in newborns have been reported, the impact of IEMs on the hospitalized newborns due to various medical conditions remains largely unknown.

The aim of this study was to estimate the incidence and characteristics of IEMs in 21,840 hospitalized neonates admitted to the Children’s Hospital of Chongqing Medical University between January 2017 and December 2022 using MS/MS and genetic analysis. The overall incidence of IEMs in hospitalized newborns was 1/377. Among the 58 cases of confirmed genetic metabolic disorders, there were 33 cases of OAMD (1/662), 23 cases of AAMD (1/950), and 2 cases of FAOD (1/10,920). In the 58 confirmed IEMs cases, a total of 72 variants was identified in 18 genes related to genetic metabolic disorders, including 23 novel variant sites, and the most commonly mutated genes were IVD, SLC25A13, and MUT. These findings enriched our understanding of the characteristics and genetics of newborn IEMs and provided screening and diagnostic insights for pediatricians.

## 2 Materials and methods

### 2.1 Patients and study design

From January 2017 to December 2022, a total of 21,840 neonates hospitalized for various diseases were screened by LC-MS/MS and/or detected by GC-MS/MS at the Children’s Hospital of Chongqing Medical University. The baseline characteristics of all infants were shown in Supplementary Table S1 (see Appendix Supplementary Table S1). The study was approved by the Ethics Committee of Children’s Hospital of Chongqing Medical University (No. 2023-248). Written informed consent was obtained from the parents or legal guardians of children.

### 2.2 LC-MS/MS and GC-MS/MS analyses

Blood was collected from the heels of newborns hospitalized for three or more days with the consent of their guardians or when medically necessary. DBS cards were prepared by placing drops of blood on special filter paper and then punching a 3.2 mm diameter of each filter paper into the U-96 wells. Samples were processed using a non-derivatized MS/MS kit (product code: 3,040-0010) (PerkinElmer, United States) according to the manufacturer’s instructions, and analyzed using the LC-MS/MS system (ABI 3200, United States). The screening indicators included 11 amino acids (including alanine (Ala), arginine (Arg), citrulline (Cit), glycine (Gly), leucine/isoleucine (Leu/Ile), methionine (Met), ornithine (Orn), phenylalanine (Phe), proline (Pro), tyrosine (Tyr), valine (Val)), and 31 acylcarnitines (including C0, C2, C3, C3DC/C4OH, C4, C4DC/C5OH, C5, C5:1, C5OH/C6OH, C6, C6DC, C8, C8:1, C10, C10:1, C10:2, C12, C12:1, C14, C14:1, C14:2, C14OH, C16, C16:1, C16:1OH, C16OH, C18, C18:1, C18:1OH, C18:2, C18OH). The cutoff values for the levels of amino acid and acylcarnitine mentioned above were presented in Supplementary Table S2 (see Appendix Supplementary Table S2).

A minimum of 5 mL of urine was collected for the GC-MS/MS analysis as previously described (Yu et al., 2021). The urine samples were treated with urease, hydroxylamine hydrochloride, sodium hydroxide and hydrochloric acid to remove urea and proteins, and 17 alkanoic acid were added as an internal standard. The samples were extracted with acetoacetate a second time, blown dry with nitrogen, and the derivatization reagent was added to a mixture of trifluoroacetamide and trimethylchlorosilane, followed by detection by GS-MS system (QP 2010, Japan) after derivatization. The cutoff values for dozens of organic acids in urine were outlined in Supplementary Table S3 (see Appendix Supplementary Table S3).

Newborns who initially showed minor abnormalities were subjected to a second sampling and retesting. Genetic analysis was performed if abnormalities were still present or if the initial preliminary results indicated a significant abnormality.

### 2.3 Genetic analyses

Following the acquisition of informed consent from guardians, 2 mL whole blood samples were collected from neonates with abnormal LC-MS/MS and/or GC-MS/MS tests, along with their parents. Exome library preparation, sequencing, bioinformatics, and data analysis were performed as previously described (Zhang et al., 2023) with minor modification. Briefly, genomic DNA (1–3 μg) was fragmented to an average size of 150 bp using a S220 Focused-ultrasonicator (Covaris, Massachusetts, United States). A DNA Sample Prep Reagent Set (MyGenostics, Beijing, China) was used for the standard libraries preparation, including end repair, adapter ligation, and PCR amplification, which would be further dual-end sequenced with a read length of 150 bp by Illumina NovaSeq 6000 system (Illumina, San Diego, CA). After sequencing, low-quality variations were filtered out using a quality score ≥20, and the clean reads were mapped to the UCSC hg19 human reference genome using the parameter BWA of Sentieon software (https://www.sentieon.com/). Variants were further annotated by ANNOVAR software (http://annovar.openbioinformatics.org/en/latest/), and associated with multiple databases, including 1000 genome, ESP6500, dbSNP, ExAC, Inhouse (MyGenostics), HGMD, and also predicted by SIFT, PolyPhen-2, MutationTaster, GERP++. The pathogenicity of identified variants was evaluated according to American College of Medical Genetics and Genomics (ACMG) guidelines. Sanger sequencing was performed to verify the identified variants.

## 3 Results

### 3.1 Schematic overview of this study

**FIGURE 1:**
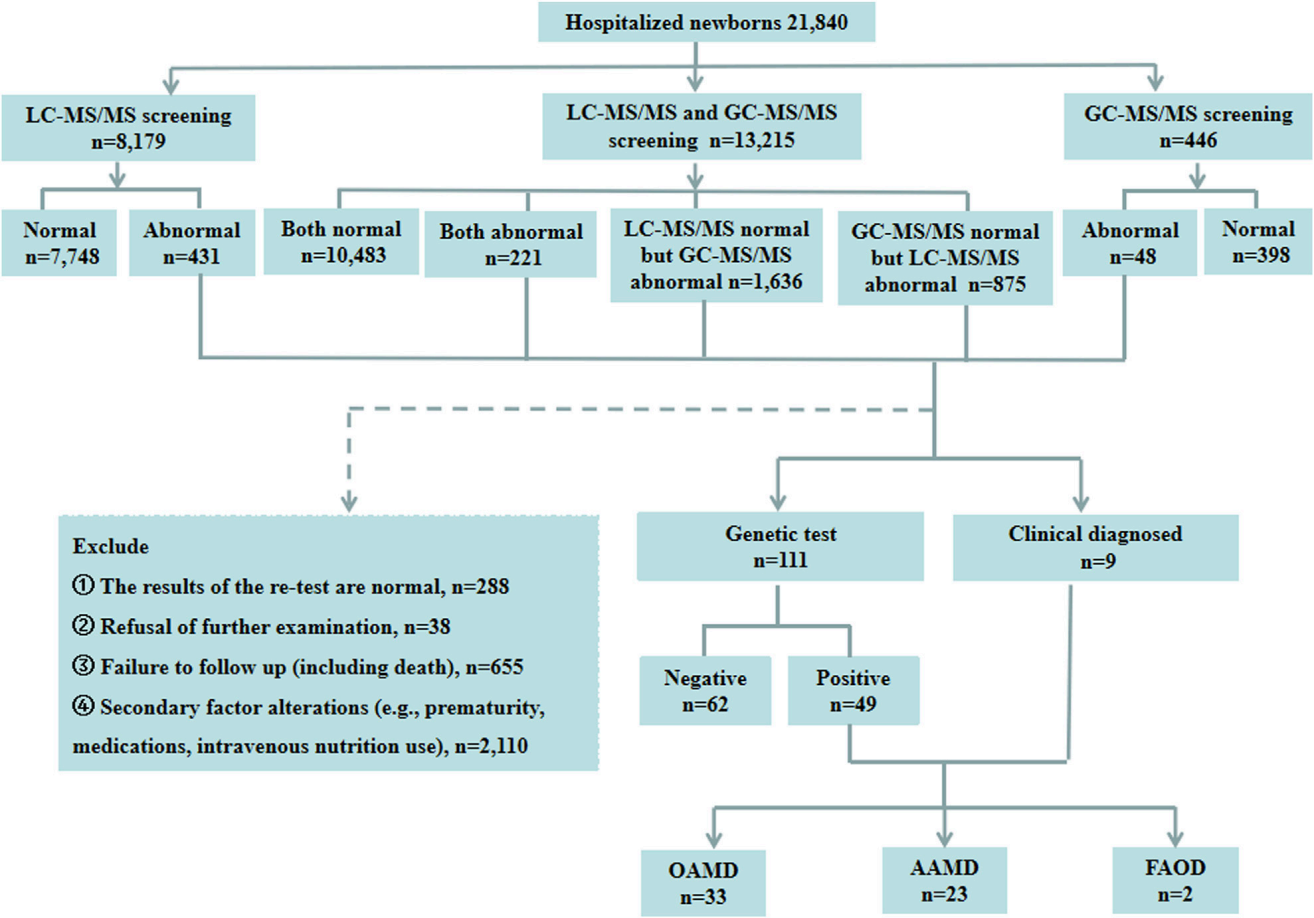
Schematic overview of the study. LC-MS/MS, liquid chromatography-tandem mass spectrometry; GC-MS/MS, gas chromatography-mass spectrometry; OAMD, organic acid metabolism disorder; AAMD, amino acid metabolism disorder; FAOD, fatty acid oxidation disorder.

A total of 21,840 hospitalized newborns were enrolled, 8,179 undergoing only LC-MS/MS, 446 undergoing only GC-MS/MS, and 13,215 undergoing both LC-MS/MS and GC-MS/MS. Among the 8,179 individuals screened only using LC-MS/MS, 7,748 exhibited normal results, and 431 showed abnormalities. Of the 446 cases subjected solely to GC-MS/MS screening, 398 were normal, and 48 were abnormal. Among the 13,215 patients who underwent both LC-MS/MS and GC-MS/MS, 10,483 had normal results for both parameters, and 221 had abnormal results for both. However, 1,636 had normal LC-MS/MS results with abnormal GC-MS/MS, and 875 had abnormal LC-MS/MS results with normal GC-MS/MS. Finally, of the 3,211 children with abnormalities detected using LC-MS/MS and/or GC-MS/MS, 111 underwent genetic testing. Of these, 49 were diagnosed with IEMs, and 62 had normal genetic test results. Two patients were directly diagnosed as IEMs based on specific LC-MS/MS indicators, while seven were diagnosed as IEMs based on specific indicators in both LC-MS/MS and GC-MS/MS. Thirty-eight cases with abnormalities in LC-MS/MS and/or GC-MS/MS declined genetic testing. The retest results of LC-MS/MS and/or GC-MS/MS were normal in 288 patients during hospitalization. Additionally, 2,765 cases were not followed up during hospitalization, including 1,755 preterm cases, 355 cases with clinically judged secondary changes such as intravenous nutrition and medications, 645 cases of refusal for followed-up, and 10 cases deaths. Of the 58 confirmed IEMs, 33 were OAMD, 23 were AAMD, and the remaining 2 cases were FAOD. The data were presented in Figure 1.

### 3.2 Incidence and composition of IEMs in hospitalized infants

**FIGURE 2:**
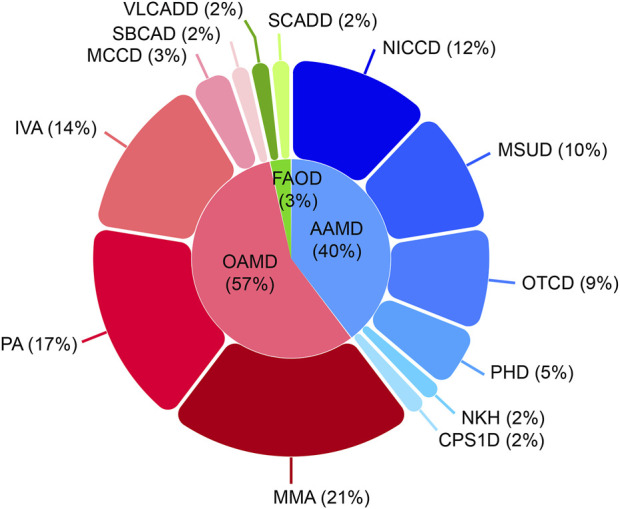
Disease spectrum and distribution of the 58 confirmed IEM cases. IEM, inborn errors of metabolism; OAMD, organic acid metabolism disorder; AAMD, amino acid metabolism disorder; FAOD, fatty acid oxidation disorder; MMA, methylmalonic acidemia; PA, propionic acidemia; IVA, isovaleric acidemia; MCCD, 3-methylcrotonyl-coenzyme A carboxylase deficiency; SBCAD, 2-methylbutanoyl-coenzyme A dehydrogenase deficiency; NICCD, neonatal intrahepatic cholestasis caused by citrin deficiency; MSUD, maple syrup urine disease; OTCD, ornithine transcarbamylase deficiency; PHD, phenylalanine hydroxylase deficiency; NKH, non-ketotic hyperglycinemia; CPS1D, carbamyl phosphate synthase I deficiency; VLCADD, very long-chain acyl-coenzyme A dehydrogenase deficiency; SCADD, short-chain acyl-coenzyme A dehydrogenase deficiency.

Among the 21,840 hospitalized infants, 58 were diagnosed with IEMs, and none of them had a history of consanguineous marriage. Preliminary statistics revealed that the incidence rate of IEMs was 1/377 (58/21,840). The disease spectra and distribution of 58 IEMs cases were shown in Table 1. Relative proportion of various IEMs were illustrated in Figure 2.

**TABLE 1:** Disease spectrum of 58 cases of genetic metabolic diseases screened from 21,840 hospitalized newborns.

Disorders(OMIM number)	Abbreviations	Confirmed cases	Incidence rate
Organic acid metabolic disorders	OAMD	33	1/662
Methylmalonic academia (#251,100 and #277,400)	MMA	12	1/1,820
Propionic acidemia (# 606,054)	PA	10	1/2,184
Isovaleric acidemia (#243,500)	IVA	8	1/2,730
3-methylcrotonyl-coenzyme A carboxylase deficiency (#210,210 and #210,200)	MCCD	2	1/10,920
2-methylbutanoyl-coenzyme A dehydrogenase deficiency (#611,283)	SBCAD	1	1/21,840
Amino acid metabolic disorders	AAMD	23	1/950
Citrin deficiency (#603,814 and #603,471)	NICCD	7	1/3,120
Maple syrup urine disease (#248,600)	MSUD	6	1/3,640
Ornithine transcarbamylase deficiency (#311,250)	OTCD	5	1/4,368
Phenylalanine hydroxylase deficiency(#261,600, and #612,349)	PHD	3	1/7,280
Non-ketotic hyperglycinemia (#238,310)	NKH	1	1/21,840
Carbamyl phosphate synthase I deficiency (#237,300)	CPS1D	1	1/21,840
Fatty acid oxidation disorders	FAOD	2	1/10,920
Short-chain acyl-coenzyme A dehydrogenase deficiency (#201,470)	SCADD	1	1/21,840
Very long-chain acyl-coenzyme A dehydrogenase deficiency (#201,475)	VLCADD	1	1/21,840

OMIM number obtained from the Online Mendelian Inheritance in Man (OMIM) database (https://omim.org/).

OAMD exhibited the highest prevalence of IEMs among hospitalized neonates, with a rate of 1/662. Within this category, 33 cases were diagnosed, including 12 cases of methylmalonic academia (MMA; 1/1,820), 10 cases of propionic acidemia (PA; 1/2,184), 8 cases of isovaleric acidemia (IVA; 1/2,730), 2 cases of 3-methylcrotonyl-coenzyme A carboxylase deficiency (MCCD; 1/10,920), and 1 case of 2-methylbutanoyl-coenzyme A dehydrogenase deficiency (SBCAD; 1/21,840).

Furthermore, 23 cases of AAMD (1/950) were identified, including 7 cases of neonatal intrahepatic cholastasis caused by citrin deficiency (NICCD; 1/3,120), 6 cases of maple syrup urine disease (MSUD; 1/3,640), 5 cases of ornithine transcarbamylase deficiency (OTCD; 1/4,368), 3 cases of phenylalanine hydroxylase deficiency (PHD; 1/7,280), 1 case of non-ketotic hyperglycinemia (NKH; 1/21,840) and 1 case of carbamyl phosphate synthase I deficiency (CPS1D; 1/21,840).

Moreover, 2 cases of FAOD (1/10,920) were found, including one of short-chain acyl-coenzyme A dehydrogenase deficiency (SCADD; 1/21,840) and one very long-chain acyl-coenzyme A dehydrogenase deficiency (VLCADD; 1/21,840).

### 3.3 Gene detection in patients with inherited metabolic disorders

Among the 58 confirmed IEM cases, 72 variant sites in 18 IEM-related genes were identified (Table 2). A total of 9 kinds of 16 mutations were detected in IVD gene: c.1199A>G, c.149G>A, c.457-3_457-2delinsGG, c.1186G>C, c.870delG, c.1231C>T, c.1123G>A, c.690_693dupTATG, and c.205G>T, with c.1199A>G being the most frequently observed mutation. A total of 7 kinds of 13 mutations have been identified in the SLC25A13 gene. Notably, c.852_855delTATG emerged as the most frequently observed mutation, alongside others such as c.1638_1660dup, c.1311 + 1G>A, c.879delT, IVS16ins3kb, c.1177 + 17C>G, and c.2006C>G. Ten different mutations were tested in 5 cases with mutations in the MMUT gene, including c.682C>T, c.1888G>A, c.1399C>T, c.1853T>C, c.788G>T, c.1703C>A, c.1777G>T, c.494A>G, c.1677G>A, and c.433G>A. Three kinds of 8 mutations were detected in the MMACHC gene, with c.609G>A being the most frequently observed mutation, along with c.567dupT and c.656-658delAGA. Five kinds of 6 mutations were identified in the PCCA gene, including c.446delA, c.524G>A, c.1145T>C, c.1102G>C, and c.1426C>T. Seven kinds of 8 mutations were found in the PCCB gene: c.898dupC, c.181C>T, c.733G>A, c.331C>T, c.838dupC, c.764G>T, and Exon 1 deletion.

**TABLE 2:** Disease spectrum, biochemical changes and genetic variants of the 58 confirmed IEM cases.

No.	Gender	Gestational week (w)	Birth weight (g)	IEM	LC-MS/MS		GC-MS/MS		Gene	Mutation 1	Mutation 2
					Abnormal parameters	Results (μmol)	Abnormal parameters	Results			
1	M	40^+2^	3,400	MSUD	Leu	4052.89	2-Keto-isocaproic-OX-2	5.85	BCKDHA	c.1000G>A (p.Gly334Arg)	c.990_993delCTAC (p.Thr331Glyfs*38)
					Val	942.93	2-Keto-3-methylpentanoic-OX-2	475.91			
							2-Keto-isovaleric-OX-2	171.93			
2	F	39^+1^	3,470	MSUD	Leu	2372.81	2-Keto-isocaproic-OX-2	303.85	BCKDHA	c.111dupC (p.Arg40Glnfs*11)	c.758C>T (p.Ala253Val)
					Val	521.68	2-Keto-3-methylpentanoic-OX-2	1.56			
							2-Keto-isovaleric-OX-2	25.97			
3	F	41	2950	MSUD	Leu	2037.57	2-Keto-isocaproic-OX-2	0.40	BCKDHB	c.670C>G (p.Leu224Val)	c.1046G>A (p.Cys349Tyr)
					Val	639.56	2-Keto-3-methylpentanoic-OX-2	189.25			
							2-Keto-isovaleric-OX-2	27.73			
4	F	39^+2^	2900	MSUD	Leu	2687.59	-		-	-	-
					Val	605.22					
5	M	38^+4^	4100	MSUD	Leu	3,899.32	-		-	-	-
					Val	612.69					
6	F	38^+2^	3,250	MSUD	Leu	2965.26	2-Keto-isocaproic-OX-2	184.03	-	-	-
					Val	524.83	2-Keto-3-methylpentanoic-OX-2	28.20			
							2-Keto-isovaleric-OX-2	15.15			
7	F	39^+5^	2400	NICCD	Cit	190.90	4-OH-phenyllactic(PHPLA)-2	16.51	SLC25A13	c.852-855delTATG (p.Met285Profs*2)	c.1638_1660dup (p.Ala554Glyfs*17)
					Met	60.87	4-OH-phenylpyruvic(PHPPA)-ox-2	1.81			
8	M	39^+4^	3,500	NICCD	Cit	522.52	4-OH-phenyllactic(PHPLA)-2	2241.21	SLC25A13	c.1311 + 1G>A (splicing)	c.852_855delTATG (p.Met285Profs*2)
					Met	551.64	4-OH-phenylpyruvic(PHPPA)-ox-2	150.23			
9	M	39^+2^	3,150	NICCD	Cit	106.47	4-OH-phenyllactic(PHPLA)-2	1188.66	SLC25A13	c.852_855delTATG (p.Met285Profs*2)	IVS16ins3kb
					Met	241.93	4-OH-phenylpyruvic(PHPPA)-ox-2	32.90			
10	M	39	2500	NICCD	Cit	77.84	4-OH-phenyllactic(PHPLA)-2	2713.41	SLC25A13	c.879delT (p.Pro293Leufs*61)	IVS16ins3kb
					Met	47.65	4-OH-phenylpyruvic(PHPPA)-ox-2	36.61			
11	M	26^+6^	1150	NICCD	Cit	8.12	4-OH-phenyllactic(PHPLA)-2	952.01	SLC25A13	c.852_855delTATG (p.Met285Profs*2)	c.1177 + 17C>G (splicing)
					Met	13.42	4-OH-phenylpyruvic(PHPPA)-ox-2	59.58			
12	F	38^+3^	2860	NICCD	Cit	14.09	4-OH-phenyllactic(PHPLA)-2	436.15	SLC25A13	c.852_855delTATG (p.Met285Profs*2)	IVS16ins3kb
					Met	41.51	4-OH-phenylpyruvic(PHPPA)-ox-2	39.00			
13	M	33 ^+6^	2240	NICCD	Cit	10.82	4-OH-phenyllactic(PHPLA)-2	2313.81	SLC25A13	c.2006C>G (p.Ser669*)	-
					Met	518.10	4-OH-phenylpyruvic(PHPPA)-ox-2	341.27			
14	F	33^+3^	1690	OTCD	Cit	5.46	Orotic-3	50.34	OTC	c.234A>G (p.Gln78Gln)	-
							Uracil-2	104.04			
15	M	39^+4^	3,500	OTCD	Cit	2.63	Orotic-3	1316.04	OTC	chrX:g.34148019_38664751del	-
							Uracil-2	19.75			
16	M	40^+1^	3,600	OTCD	Cit	3.50	Orotic-3	366.63	-	-	-
							Uracil-2	18.26			
17	M	40^+1^	3,040	OTCD	Cit	2.79	Orotic-3	161.89	OTC	c.1048C>T (p.Gln350*)	
							Uracil-2	0			
18	M	36^+6^	2880	OTCD	Cit	2.45	Orotic-3	573.34	-	-	-
							Uracil-2	24.19			
19	F	38^+4^	2350	CPS1D	Cit	4.31	Normal		CPS1	c.381+1delG (splicing)	c.1754T>C (p.Met585Thr)
20	M	37^+4^	2350	PHD	Phe	192.77	-		PAH	c.611A>G (p.Tyr204Cys)	c.158G>A (p.Arg53His)
					Phe/Tyr	5.94					
21	F	38^+2^	2980	PHD	Phe	268.21	-		PAH	c.611A>G (p.Tyr204Cys)	c.505C>T (p.Arg169Cys)
					Phe/Tyr	4.80					
22	M	39^+3^	3,300	PHD	Phe	574.21	Normal		PAH	c.721C>T (p.Arg241Cys)	c.441 + 3G>C
					Phe/Tyr	5.87					
23	M	39^+4^	3,210	NKH	Gly	1843.30	-		AMT	c.887G>A (p.Arg296His)	c.826G>C (p.Asp276His)
24	F	39^+1^	3,880	IVA	C5	4.78	3-OH-isovaleric-2	143.68	IVD	c.1199A>G (p.Tyr400Cys)	c.1199A>G (p.Tyr400Cys)
					C5/C2	2.62	Isovalerylglycine-1	118.40			
							Isovalerylglycine-2	529.90			
25	F	39^+4^	3,290	IVA	C5	6.70	3-OH-isovaleric-2	379.12	IVD	c.149G>A (p.Arg50His)	c.1199A>G (p.Tyr400Cys)
					C5/C2	1.33	Isovalerylglycine-1	1533.50			
							Isovalerylglycine-2	1.18			
26	F	39^+1^	2700	IVA	C5	5.64	3-OH-isovaleric-2	217.55	IVD	c.457-3_457-2delinsGG	c.1186G>C (p.Asp396His)
					C5/C2	1.12	Isovalerylglycine-1	964.11			
							Isovalerylglycine-2	162.03			
27	F	39^+2^	3,350	IVA	C5	6.59	3-OH-isovaleric-2	506.35	IVD	c.149G>A (p.Arg50His)	c.1199A>G (p.Tyr400Cys)
					C5/C2	0.98	Isovalerylglycine-1	282.00			
							Isovalerylglycine-2	1130.41			
28	M	39	2580	IVA	C5	5.12	3-OH-isovaleric-2	0	IVD	c.870delG (p.Pro291leufs*18)	c.1231C>T (p.Arg411Trp)
					C5/C2	2.05	Isovalerylglycine-1	651.44			
							Isovalerylglycine-2	622.96			
29	M	39^+4^	3,400	IVA	C5	8.18	-		IVD	c.1123G>A (p.Gly375Ser)	c.1186G>C (p.Asp396His)
					C5/C2	3.49					
30	M	39	3,500	IVA	C5	5.71	-		IVD	c.690_693dupTATG (p.Pro232Tyrfs*5)	c.1199A>G (p.Tyr400Cys)
					C5/C2	2.59					
31	M	39^+3^	3,315	IVA	C5	7.32	-		IVD	c.1199A>G (p.Tyr400Cys)	c.205G>T (p.Asp69Tyr)
					C5/C2	0.19					
32	M	39^+1^	2755	MMA	C3	6.59	Methylmalonic-2	39.12	MMACHC	c.609G>A (p.Trp203*)	c.609G>A (p.Trp203*)
					C3/C2	0.95	Methylcitric-4(2)	1.92			
33	F	40^+2^	2710	MMA	C3	7.80	Methylmalonic-2	153.42	MMACHC	c. 567dupT (p.Ile190TyrfsTer13)	c. 567dupT (p.Ile190TyrfsTer13)
					C3/C2	1.25	Methylcitric-4(2)	7.26			
34	M	39^+4^	2200	MMA	C3	3.92	Methylmalonic-2	1827.00	MMACHC	c.656-658delAGA (p.Lys220Argfs*71)	c.609G>A (p.Trp203*)
					C3/C2	0.63	Methylcitric-4(2)	38.00			
35	M	41^+2^	3,000	MMA	C3	3.71	Methylmalonic-2	126.00	MMACHC	c.609G>A (p.Trp203*)	c.656-658delAGA (p.Lys220Argfs*71)
					C3/C2	0.95	Methylcitric-4(2)	0			
36	M	39^+2^	3,150	MMA	C3	9.70	Methylmalonic-2	99.88	MUT	c.682C>T (p.Arg228*)	c.1888G>A (p.G630R)
					C3/C2	0.64	Methylcitric-4(2)	14.44			
37	M	39^+6^	2750	MMA	C3	5.79	Methylmalonic-2	1370.00	MUT	c.1399C>T (p.Arg467*)	c.1853T>C (p.Leu618Pro)
					C3/C2	1.48	Methylcitric-4(2)	29.37			
38	F	37^+6^	2600	MMA	C3	44.38	Methylmalonic-2	106.32	MUT	c.788G>T (p.Gly263Val)	c.1703C>A (p.Ala568Asp)
					C3/C2	2.02	Methylcitric-4(2)	12.85			
39	F	39^+2^	2900	MMA	C3	13.17	Methylmalonic-2	265.85	MUT	c.1777G>T (p.Glu593*)	c.494A>G (p.Asp165Gly)
					C3/C2	0.79	Methylcitric-4(2)	5.41			
40	F	39^+2^	2900	MMA	C3	10.64	-		MUT	c.1677-1G>A (splicing)	c.433G>A (p.Gly145Ser)
					C3/C2	0.75					
41	F	39^+2^	2450	MMA	C3	9.27	Methylmalonic-2	929.78	MMAB	c.556C>T (p.Arg186Trp)	c.581_582delTC (p.Arg194Thrfs*24)
					C3/C2	0.78	Methylcitric-4(2)	19.36			
42	M	33^+5^	2000	MMA	C3	9.23	Methylmalonic-2	1735.80	-	-	-
					C3/C2	2.10	Methylcitric-4(2)	9.46			
43	F	39 + 4	3,200	MMA	C3	1.48	Methylmalonic-2	253.87	ACSF3	c.628A>C (p.Lys210Gln)	c.1084A>T (p.Met362Leu)
					C3/C2	0.09					
44	M	40^+5^	3,400	PA	C3	14.50	3-OH-propionic-2	2235.73	PCCB	c.898dupC (p.Leu300Profs*11)	Exon 1 deletion
					C3/C2	1.86	3-Methylcrotonylglycine-1	89.96			
45	F	40^+2^	3,450	PA	C3	36.33	3-OH-propionic-2	20.31	PCCB	c.181C>T (p.Arg61*)	c.733G>A (p.Gly245Ser)
					C3/C2	3.80	3-Methylcrotonylglycine-1	4.89			
46	F	40^+4^	4150	PA	C3	12.26	3-OH-propionic-2	127.64	PCCB	c.331C>T (p.Arg111*)	c.838dupC (p.Leu280Profs*11)
					C3/C2	7.63	3-Methylcrotonylglycine-1	0.47			
47	F	39^+2^	3,130	PA	C3	16.66	3-OH-propionic-2	212.19	PCCB	c.838dupC (p.Leu280Profs*11)	c.764G>T (p.Gly255Val)
					C3/C2	1.23	3-Methylcrotonylglycine-1	3.22			
48	M	39^+5^	3,760	PA	C3	11.61	-		PCCA	c.524G>A (p.Gly175Asp)	c.1145T>C (p.Leu382Pro)
					C3/C2	1.09					
49	F	40^+2^	3,940	PA	C3	7.44	-		PCCA	c.446delA (p.Asn149fs)	c.1102G>C (p.Asp368His)
					C3/C2	1.90					
50	F	38^+5^	3,600	PA	C3	14.16	3-OH-propionic-2	0.72	PCCA	c.446delA (p.Asn149fs)	c.1426C>T (p.Arg476Ter)
					C3/C2	2.41	3-Methylcrotonylglycine-1	1.15			
51	M	37^+2^	2520	PA	C3	14.11	3-OH-propionic-2	205.65	-	-	-
					C3/C2	1.80	3-Methylcrotonylglycine-1	5.36			
52	M	38^+1^	3,415	PA	C3	9.03	3-OH-propionic-2	396.59	-	-	-
					C3/C2	2.61	3-Methylcrotonylglycine-1	8.05			
53	M	40^+1^	3,750	PA	C3	10.79	3-OH-propionic-2	643.78	-	-	-
					C3/C2	3.63	3-Methylcrotonylglycine-1	18.79			
54	M	32^+6^	1850	MCCD	C5-OH	1.80	3-Methylcrotonylglycine-1	74.05	MCCC1	c.1331G>A (p.Arg444His)	c.1252A>C (p.Thr418Pro)
							3-OH-isovaleric-2	2432.10			
55	M	33^+6^	2460	MCCD	C5-OH	8.27	3-Methylcrotonylglycine-1	34.77	MCCC1	c.1679_c.1680insA (p.Asn560Lysfs*10)	c.1679_c.1680insA (p.Asn560Lysfs*10)
							3-OH-isovaleric-2	807.47			
56	F	40^+6^	3,300	SBCAD	C5	0.43	Normal		ACADSB	c.1165A>G (p.Met389Val)	c.1165A>G (p.Met389Val)
					C5/C2	0.04					
57	F	38^+4^	3,700	VLCAD	C14:1	11.08	Adipic	34.77	ACADVL	c.1843C>T (p.Arg615Ter)	c.1346A>C (p.Glu449Ala)
					C14	4.21	Suberic-2	25.05			
58	F	37^+4^	2480	SCADD	C4	1.53	-		ACADS	c.1148G>A (p.Arg383His)	c.1031A>G (p.Glu344Gly)
					C4/C2	0.12					

M, male; F, female; IEM, inborn errors of metabolism; LC-MS/MS, liquid chromatography-tandem mass spectrometry; GC-MS/MS, gas chromatography-mass spectrometry; MSUD, maple syrup urine disease; NICCD, neonatal intrahepatic cholestasis caused by citrin deficiency; OTCD, ornithine transcarbamylase deficiency; CPS1D, carbamyl phosphate synthase I deficiency; PHD, phenylalanine hydroxylase deficiency; NKH, non-ketotic hyperglycinemia; IVA, isovaleric acidemia; MMA, methylmalonic acidemia; PA, propionic acidemia; MCCD, 3-methylcrotonyl-coenzyme A carboxylase deficiency; SBCAD, 2-methylbutanoyl-coenzyme A dehydrogenase deficiency; VLCADD, very long-chain acyl-coenzyme A dehydrogenase deficiency; SCADD, short-chain acyl-coenzyme A dehydrogenase deficiency.

Of all the genetic variants detected, a total of 23 mutations in 18 genes had not been previously reported, including 12 missense mutations (such as c.1000G>A and c.758C>T in the BCKDHA gene, c.670C>G in the BCKDHB gene, c.628A>C and c.1084A>T in the ACSF3 gene, c.1754T>C in the CPS1 gene, c.1888G>A and c.1703C>A in the MMUT gene, c.764G>T in the PCCB gene, c.1145T>C in the PCCA gene, c.1346A>C in the ACADVL gene, c.1252A>C in the MCCC1 gene), four frameshift mutations (including c.879delT in the SLC25A13 gene, c.898dupC in the PCCB gene, c.1679_c.1680insA in the MCCC1 gene and c.990_993delCTAC in the BCKDHA gene), three termination mutations (including c.1048C>T in the OTC gene, c.181C>T in the PCCB gene and c.2006C>G in the SLC25A13 gene), a shear mutation (c.381+1delG in the CPS1 gene), a synonymous mutation (c.234A>G in the OTC gene), an intronic mutation (c.1177 + 17C>G in the SLC25A13 gene), and a large 4.52 Mb hemizygous deletion containing the OTC gene (seq[hg19]del(X) (p21.1p11.4) chrX:g.34148019_38664751del).

The pathogenicity of the 23 previously unreported variants mentioned above was analyzed using the ACMG rating system. Three mutations [a large 4.52 Mb hemizygous deletion containing the OTC gene (seq[hg19]del(X)(p21.1p11.4)chrX:g.34148019_38664751del), c.898dupC in the PCCB gene and c.1679_c.1680insA in the MCCC1 gene] were identified as pathogenic and 6 mutations (c.1000G>A and c.990_993del CTAC in the BCKDHA gene, c.879delT in the SLC25A13 gene, c.381+1delG in the CPS1 gene, c.1888G>A in the MMUT gene, and c.181C>T in the PCCB gene) were identified as likely pathogenic. The remainder were of uncertain significance, of which four were reported with different amino acid substitutions at the same position, three variants were indexed in the Clinvar database lacked relevant literature, and the remaining seven unreported variants of unknown significance included a synonymous mutation, two termination mutations, three missense mutations and one intronic variant. The data were shown in Table 3.

**TABLE 3:** The 23 previously unreported variants of 11 genes in patients with IEMs in this study.

IEM	Gene	Chr	Position	cDNA	Protein	Exon	ACMG	Evidence
MSUD	BCKDHA	19	41928907	c.1000G>A	p.G334R	8	LP	PM2_Supporting, PM5, PP3_Strong
		19	41928180	c.758C>T	p.A253V	6	US	PM2_Supporting, PM5, PP3_Moderate
		19	41928668	c.990_993delCTAC	p.T331Gfs*38	7	LP	PVS1, PM2_Supporting
	BCKDHB	6	80881035	c.670C>G	p.L224V	6	US	PM2_Supporting, PP3_Moderate
NICCD	SLC25A13	7	95818659–95818660	c.879delT	p.P293Lfs*61	9	LP	PVS1, PM2_Supporting
		7	95813572	c.1177+17C>G	Splicing	11	US	BP4
		7	95750528	c.2006C>G	p.S669*	18	US	PVS1_Moderate, PM2_Supporting
OTCD	OTC	X	38229066	c.234A>G	p.Q78Q	3	US	PM2_Supporting
		X	38280318	c.1048C>T	p.Q350*	10	US	PVS1_Moderate, PM2_Supporting
		X	34148019–38664751	chrX:g.34148019_38664751del	NA	NA	P	1A(0) + 2A(1) + 3A(0) = 1
CPS1D	CPS1	2	211441214	c.381+1delG	Splicing	3	LP	PVS1, PM2_Supporting
		2	211466972	c.1754T>C	p.M585T	16	US	PM2_Supporting, PP3_Strong
MMA	MMUT	6	49409658	c.1703C>A	p.A568D	10	US	PM2_Supporting, PP3_Strong
		6	49407987	c.1888G>A	p.G630R	11	LP	PM2_Supporting, PM5, PP3_Strong
	ACSF3	16	89167717	c.628A>C	p.K210Q	3	US	PP3_Strong
		16	89180853	c.1084A>T	p.M362L	6	US	PM2_Supporting
PA	PCCB	3	136016865	c.898dupC	p.L300Pfs*11	8	P	PVS1, PM3_Strong, PM2_Supporting
		3	135969398	c.181C>T	p.R61X	1	LP	PVS1, PM2_Supporting
		3	136016794	c.764G>T	p.G255V	8	US	PM2_Supporting, PP3_Strong
	PCCA	13	100953793	c.1145T>C	p.L382P	13	US	PM2_Supporting, PP3_Strong
MCCD	MCCC1	3	182759370	c.1252A>C	p.T418P	11	US	PM2_Supporting, PP3_Strong
		3	182751780–182751781	c.1679_1680insA	p.N560Kfs*10	14	P	PVS1, PM2_Supporting, PM3_Strong
VLCAD	ACADVL	17	7127300	c.1346A>C	p.E449A	14	US	PM2_Supporting, PP3_Strong

IEM, inborn errors of metabolism; Chr, Chromosome; MSUD, maple syrup urine disease; NICCD, neonatal intrahepatic cholestasis caused by citrin deficiency; OTCD, ornithine transcarbamylase deficiency; CPS1D, carbamyl phosphate synthase I deficiency; MMA, methylmalonic acidemia; PA, propionic acidemia; MCCD, 3-methylcrotonyl-coenzyme A carboxylase deficiency; VLCADD, very long-chain acyl-coenzyme A dehydrogenase deficiency; NA, no data available; P, pathogenic; LP, likely pathogenic; US, uncertain significance.

### 3.4 Clinical biochemistry status, clinical presentations and prognosis of children diagnosed with IEMs

**FIGURE 3:**
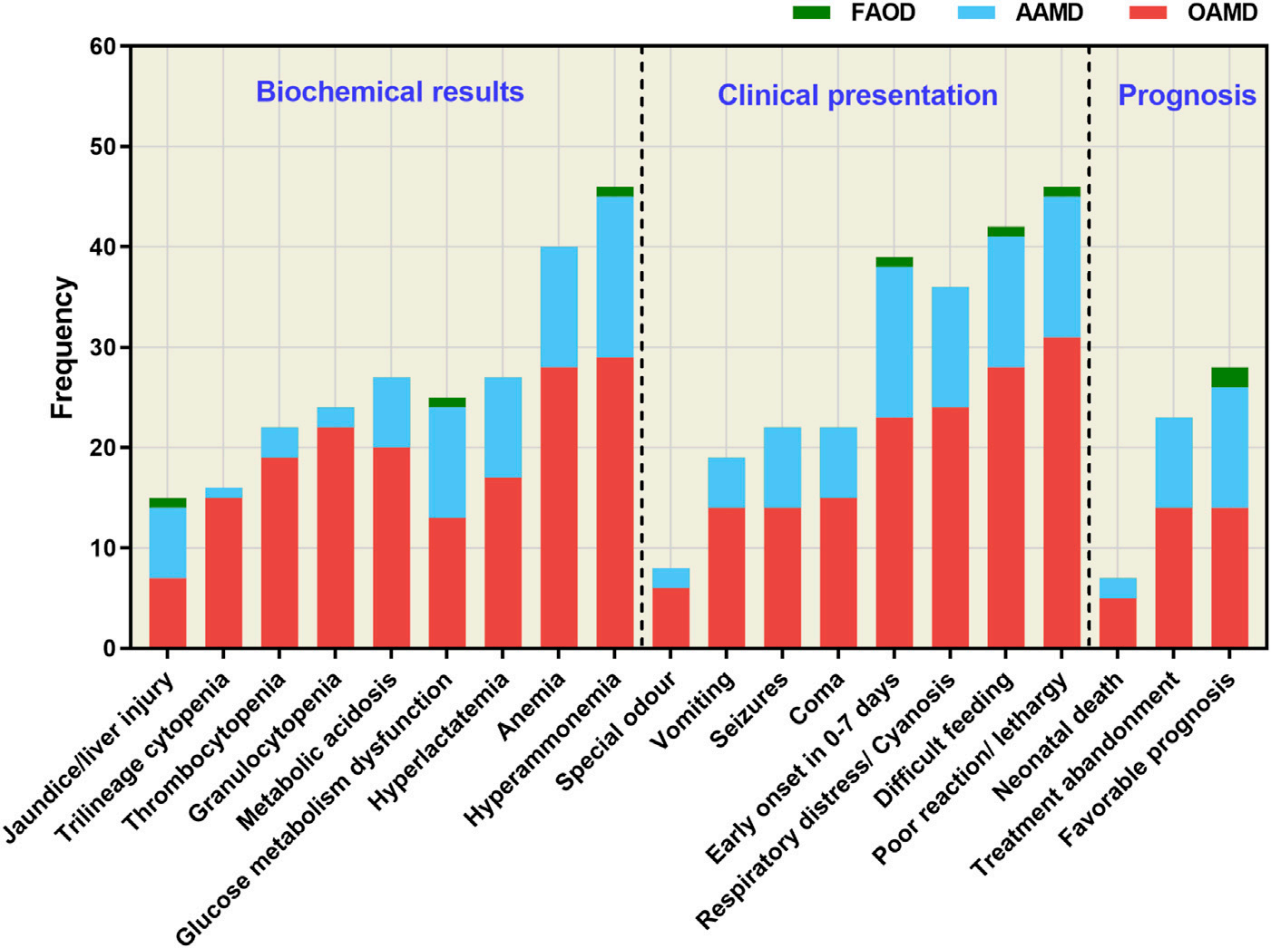
Clinical biochemistry, clinical manifestations, and prognosis of the 58 confirmed IEM cases. FAOD, fatty acid oxidation disorder; AAMD, amino acid metabolism disorder; OAMD, organic acid metabolism disorder.

To assess the clinical biochemistry status, clinical manifestations, and prognosis of 58 confirmed IEMs, we analyzed the data shown in Figure 3. Among these cases, 39 had onset within the first 7 days after birth. The primary clinical features included poor response/somnolence, feeding difficulty, respiratory distress/cyanosis, coma, seizures, vomiting and other associated symptoms. Biochemical abnormalities were mainly characterized by hyperammonemia, anemia, glucose abnormalities, metabolic acidosis, hyperlactacidemia, thrombocytopenia, cholestasis/liver injury, leukopenia and anemia. Notably, clinical phenotypes and biochemical indices were more pronounced at disease onset in children with OAMD.

Of the 58 children diagnosed with IEMs, 23 had to discontinue treatment due to the severity of their conditions, including 14 cases of OAMD (6 cases of MMA, 5 cases of PA and 3 cases of IVA), as well as 9 cases of AAMD (5 cases of MSU, 2 cases of OTCD, 1 case of CPS1D and 1 case of NKH). Unfortunately, 7 cases resulted in neonatal mortality, including 5 cases of OAMD (3 cases of IVA and 2 cases of MMA) and 2 cases of AAMD (2 cases of OTCD). Notably, 28 cases maintained long-term survival following timely diagnosis and treatment, including 12 cases of AAMD (7 cases of NICCD, 3 cases of PKU, 1 case of OTCD, and 1 case of MSUD), 14 cases of OAMD (5 cases of PA, 4 cases of MMA, 2 cases of MCCD, 2 cases of IVA, and 1 case of SBCAD) and 2 cases of FAOD (1 case of SCADD and 1 case of ACADVLD).

PKU, as an important disease for newborn screening, was found in three cases in our study. Among them, 2 infants (case 20 and case 21 in Table 2) were diagnosed with mild hyperphenylalaninemia and were followed up regularly by monitoring blood phenylalanine concentrations. Their blood phenylalanine concentrations were regularly monitored, fluctuating between 2 and 3 mg/dL. One infant (case 22 in Table 2) was diagnosed with classic phenylketonuria and a low-phenylalanine diet was maintained. Follow-up has been conducted for 1 year and 3 months to date. The blood phenylalanine concentrations remained within the ideal range (2–4 mg/dL) and the growth and development of this child, including height, weight, and cognitive function, were comparable to those of peers of the same age. None of the three cases showed neurological damage due to elevated phenylalanine concentrations.

## 4 Discussion

The incidence of IEMs in hospitalized neonates displayed considerable variation across different studies. In our investigation of 21,840 neonates hospitalized for various medical conditions, the incidence of IEMs was 1/377. This rate surpassed that of high-risk neonates in Liuzhou, China (1/393) (Tan et al., 2021) and in the NICU in Beijing, China (1/640) (Zhang et al., 2020), but was lower than the incidence reported in Spain (1/113), where the rate in NICU neonates reached 1.63% (Couce et al., 2011). The Children’s Hospital of Chongqing Medical University, ranked as the third specialized children’s hospital in China, serves as an important diagnosis and treatment center for high-risk neonates with a proportion of about 40% of patients from other cities. This may explain the high incidence of IEMs observed in the present study. Within our study cohort, 67.2% (39/58) of the patients with IEMs presented with clinical symptoms and/or biochemical abnormalities indicative of metabolic imbalances within the first 7 days of life. Among the 58 identified cases of IEMs, 56.9% (33 cases) were OAMD (MMA, PA, IVA, MCCD, SCAD), 39.7% (23 cases) were AAMD (NICCD, MUSD, NKH, OTCD, PHD, CPS1D), and 3.4% (2 cases) were FAOD.

OAMD was the predominant category of IEMs in hospitalized newborns in our study, with MMA exhibiting the highest incidence rate. The global incidence of MMA varied greatly, registering approximately 1/81,967 in Europe, 1/16,556 in the Middle East and North Africa (Almási et al., 2019), and 1/36,376 in Japan (Yamaguchi, 2008). Notably, the incidence in China was higher, yet substantial regional disparities were evident. For instance, the incidence of MMA was about 1/3,220 in Jining (Guo et al., 2018), 1/16,833 in Jiangsu Xuzhou district (Zhou et al., 2019), 1/46,531 in Zhejiang province (Hong et al., 2017), and 1/6,264 in Xinxiang (Ma et al., 2020). In our study, 12 confirmed cases of MMA resulted in an incidence rate of 1/1,820, which was significantly higher than the incidence observed in high-risk children in Liuzhou, China (1/7,461) (Tan et al., 2021). Clinical investigations have indicated an increasing global incidence of MMA, evolving from 1/81,967 a decade ago to around 1/10,660. In southern China, the incidence of MMA ranged from approximately 1/86,000 to 1/46,500, and in the north, it varied from about 1/26,000 to 1/6,032. Interestingly, there was a decline from 1/97 to 1/256 in clinically suspected patients (Jin et al., 2021). Among the 11 genetically confirmed MMA cases in our study, five were MUT type, four were MMACHC type, one was MMAB type, and one was ACSF3 type. The distribution of disease types deviated from the prevalent MMACHC type in the Chinese population, which was consistent with the findings reported by Zhang (Zhang et al., 2020) and suggested that isolated types were more common during the acute neonatal period. PA, an autosomal recessive disorder mainly caused by defects in the PCCA or PCCB genes, was the second most common form of OAMD in hospitalized neonates. Although the worldwide incidence of PA ranged from 1/100,000 to 1/50,000 (Shibata et al., 2018), no national date was reported in China. In our study, the prevalence of PA was approximately 1/2,184. PA commonly occurred during the neonatal period with symptoms such as poor feeding, vomiting, and hyperammonemia. The differentiation of PA can be facilitated through elevated C3 levels detected by LC-MS/MS and elevated 3-OH-propionic-2 levels by GC-MS/MS. IVA was the third most prevalent disease of OAMD. The incidence of IVA varied significantly among different regions and populations, with approximately 1/45,466 (Scolamiero et al., 2015) in Italy, 1/33,282 in the Middle East (Golbahar et al., 2013) and 1/265,900 in Zhejiang, China (Hong et al., 2017). However, the prevalence spiked significantly in hospitalized neonates or high-risk groups, reaching around 1/7,461 in Quanzhou, China, and was notably higher (1/2,754) in our study.

AAMD, encompassing conditions such as NICCD, MSUD, and OTCD, were the second most common IEMs identified in hospitalized neonates in our study. Among these, NICCD stood out as the most common AAMD, with an incidence rate of 1/3,120. The occurrence of NICCD exhibited noteworthy regional variations within China, being notably higher in the south than in the north. NICCD primarily manifested in neonates or infants, and was characterized by delayed resolution of jaundice, cholestasis and coagulation disorders. The disease prognosis was generally favorable, with most cases resolving by the age of 1 year. Although early screening or diagnosis of NICCD predominantly relied on the elevated citrulline levels, which represented the earliest detectable metabolite change, relying solely on citrulline levels tended to lead to missed diagnoses. Alternatively, some patients exhibited almost normal levels of amino acids such as citrulline and methionine, in the neonatal period. Hence, not all instances of NICCD can be identified early by neonatal disease screening (Lin et al., 2019; Lin et al., 2023). In our study, 3 patients with NICCD displayed normal citrulline levels during biochemical changes such as cholestasis, jaundice, and hepatic impairment. However, these patients showed significant increases in the excretion of 4-Hydroxyphenolactic acid and 4-Hydroxyphenylpyruvic acid, eventually confirming the diagnosis of NICCD through genetic testing. MSUD ranked as the second most prevalent AAMD, and was characterized by rapid onset, quick progression, and unfavorable prognosis. Globally, MSUD has been reported to have an incidence of 1/185,000 (Jiang et al., 2021). In our study, we identified 6 cases of MSUD with an incidence of approximately 1/3,540. Due to the severity of the disease, 5 patients discontinued treatment, which may be the reason there were no reported data on its incidence. Specific markers for MSUD include elevated levels of leucine, isoleucine, and valine in the blood. In our cohort, all 6 patients exhibited significantly elevated levels of leucine and valine, as detected by LC-MS/MS. However, only 3 cases underwent genetic testing, while the remaining 3 cases were diagnosed directly based on LC-MS/MS findings and clinical symptoms. OTCD was the most common type of congenital urea cycle disorder, and was an X-linked incomplete dominant genetic disease that affected males carrying the dominant gene as homozygous. Heterozygous female patients may remain asymptomatic or experience milder symptoms than male patients. The impact of the disease primarily targeted brain development, and the symptoms varied in severity. Onset during the neonatal period was usually rapid, particularly in male patients, emphasizing the importance of timely diagnosis and treatment (Crowe et al., 2018). In our study, four of five male neonates diagnosed with OTCD died after treatment was terminated due to the severity of their condition.

As a pivotal method in the screening and diagnosis of IEMs, LC-MS/MS plays a vital role in detecting abnormal metabolites in DBS. However, LC-MS/MS encounters several challenges, including the large amount of data generate and the susceptibility of certain metabolites to various factors, such as birth weight, gestational age, medication, venous nutrition, and late-onset, leading to potential false positives or false negatives. For instance, individuals with late-onset OTCD, especially female heterozygotes and MMA, may remain asymptomatic until they are triggered by external factors such as infections, surgeries, vaccinations, and high protein intake. In our patient cohort, 2 cases of MMA and 1 case of malonic acid-methylmalonic aciduria exhibited normal C3 levels and associated ratios during early hospitalization. The observed variation could be attributed to genotype and individual differences. Moreover, IEMs usually exhibit significant disparities in metabolite accumulation across different periods and individuals. Different types of IEMs can present with abnormalities in the same amino acid or acylcarnitine. For example, both OTCD and CPS1D showed a decrease in citrulline, whereas MMA and PA showed an increase in C3, C3/C0 and C3/C2 levels. Thus, diagnosis based solely on abnormal blood metabolites is not advisable, and further diagnosis is often necessary, involving the consideration of abnormal metabolites in urine, especially for IEMs with specific urinary metabolites. The presence or absence of the metabolite methylmalonic acid in the urine served as a distinguishing factor between MMA (positive) and PA (negative) patients. Additionally, both OTCD and CPS1D patients may have reduced citrulline levels in the blood. However, patients with OTCD may exhibit significantly elevated Orotic-3 levels in the urine, whereas CPS1D patients have either normal or low Orotic-3 levels. This distinction allowed for the differentiation between the two diseases based on the Orotic-3 level in the urine. Therefore, the integration of LC-MS/MS and GC-MS/MS is an optimal and comprehensive strategy for efficient screening and diagnosis of IEMs (Patial et al., 2022).

The complexity arising from phenotypic variations within the same disease and the similarities between distinct diseases posed considerable challenges in diagnosing IEMs. High-throughput sequencing technology has emerged as a pivotal tool to address this challenge. In our study, 49 cases of IEMs were diagnosed through genetic testing, including 6 cases with normal LC-MS/MS results but significantly abnormal GC-MS/MS results. Among these cases, 72 variants in 18 genes associated with IEMs were detected, with 23 variants not previously reported, highlighting the significance of ongoing exploration in the field.

Among the diagnosed OAMD, 5 cases of MMA with the MMUT gene were identified, revealing 10 mutations, including c.1888G>A and c.1703C>A, which have not been reported previously. Among the 4 cases of MMACHC-type MMA, three different mutations were detected, with c.609G>A emerging as the most common, which was consistent with findings in other domestic reports (Wang et al., 2010). The incidence of MMA with the MMAB and ACSF3 genes was low and has rarely been reported. Mutations c.1888G>A and c.1703C>A in the MMUT gene, as well as c.628A>C and c.1084A>T in the ACSF3 gene, have not been previously documented.

Among the 7 cases diagnosed with PA, four had variants in the PCCB gene, and the remaining three exhibited variants in the PCCA gene, with no hotspot mutations identified. Moreover, the c.1145T>C mutation in the PCCA gene and the c.764G>T, c.898dupC and c.181C>T mutations in the PCCB gene have not been previously reported. Interestingly, c.922_923insT, c.1644–6C>G, and c.1196G>A were the main mutations in the PCCA gene in Japanese patients with PA, while c.1283C>T and c.1228C>T were the predominant mutations in the PCCB gene (Yang et al., 2004). The c.1218_1231del14ins12 mutation in the PCCB gene was the most common among Caucasians (Campeau et al., 1999). However, hotspot mutations in the PCCA and PCCB genes have not yet been reported in China.

In 8 cases of IVA, 11 types of 16 mutations in the IVD gene were identified. Among them, the c.1199A>G mutation was the most frequently detected, occurring in 5 cases. The c.932C>T mutation, which was recognized as a hotspot mutation in Germany and the United States (Ensenauer et al., 2004), was also observed. Conversely, the c.1208A>G mutation, identified as a high-frequency mutation in China (Li et al., 2019), was detected only twice in this study. This may be related to the smaller population size and regional differences. The three most prevalent AAMD observed in hospitalized neonates were MSUD, NICCD, and OTCD. Of the 6 cases diagnosed with MSUD, only three received definitive diagnoses by genetic testing. Currently, there were only scattered case reports in China, and no identified gene variants and frequencies have been reported. Among the 3 cases of MSUD subjected to genetic testing, 2 cases exhibited mutations in the BCKDHA gene, while the remaining one had a mutation in the BCKDHB gene (Table 2). Notably, the c.758C>T, c.1000G>A, and c.990_993delCTAC mutations found in the BCKDHA gene have not been previously reported, and there were no available data on the c.670C>G mutation in the BCKDHB gene. Among the seven NICCD cases, 13 mutation sites and seven mutation types were detected, with the c.852_855delTATG mutation site showing the highest frequency. Currently, various gene variants of the SLC25A13 gene have been identified, including c.852_855delTATG, c.1638_1660dup, IVS6+5G>A, and IVS16ins3kb, which collectively accounted for over 80% of all variations in the Chinese population (Lin et al., 2016).

In addition, we analyzed 23 previously unreported genetic variants and evaluated their pathogenicity using the ACMG rating system. Among these, 3 variants were classified as pathogenic: a large 4.52 Mb hemizygous deletion containing the OTC gene (seq[hg19]del(X) (p21.1p11.4)chrX:g.34148019_38664751del), c.898dupC in the PCCB gene and the c.1679_c.1680insA mutation in the MCCC1 gene. Furthermore, 6 variants were rated as likely pathogenic: c.1000G>A and c.990_993delCTAC in the BCKDHA gene, c.879delT in the SLC25A13 gene, c.381+1delG in the CPS1D, c.1888G>A in the MMUT gene, and c.181C>T in the PCCB gene. The remaining 14 unreported variants were categorized as uncertain significance by the ACMG system. Using the Swiss-model web tool (http://swissmodel.expasy.org/interactive), we employed predictive analysis to assess the changes in the three-dimensional structure of proteins resulting from these missense mutations regarded as uncertain significance. The c.1252A>C mutation in the MCCC1 gene results in a switch from threonine to proline at the 418th amino acid position of the encoded protein. This alteration was predicted to cause the loss of threonine’s ability to form hydrogen bonds with other amino acids at the 418th position, potentially influencing the conformation of the protein. This phenomenon occurs because, in the absence of this mutation, threonine at position 418 can establish three hydrogen bonds with valine at position 420 and isoleucine at position 434. The c.670C>G mutation in the BCKDHB gene, the c.1703C>A mutation in the MMUT gene, and the c.764G>T mutation in the PCCB gene resulted in a transition from one amino acid to another, with no change in the number of hydrogen bonds between amino acids. Consequently, the effect of these mutations on the spatial structure of the protein remains unclear. Hence, the implications of these mutations on the spatial structure of the protein have not been definitively established, and further functional validation is imperative to elucidate their pathogenicity.

Among the 21,840 hospitalized neonates, 41.05% (8,966/21,840) were preterm, and 58.95% (12,874/21,840) were full-term infants. Of the 8,966 preterm infants, seven were diagnosed with IEMs, with a prevalence of 1/1,281. This contrasted with 51 cases of IEMs identified in 12,874 full-term babies, yielding a prevalence of 1/248, which was 5 times higher than that in preterm infants and similar to a Spanish study (Martín-Rivada et al., 2022). It is possible that maternal and placental metabolism confer a protective effect on fetuses with IEMs. These infants were susceptible to metabolic toxicity, such as hyperammonemia and metabolic acidosis, within days or even hours after birth, due to exposure to external factors such as the environment, feeding, and vaccination. Patients with OAMD appeared to be more susceptible to hematological complications, including anemia, thrombocytopenia, and leukopenia, especially those with MMA, IVA, and PA. Toxic metabolic byproducts can accumulate in multiple systems, affecting the nervous and hematopoietic systems and resulting in diverse clinical and biochemical abnormalities. Previous studies have also found that children with OAMD may develop anemia, neutropenia, or thrombocytopenia during acute exacerbations, possibly because of abnormal metabolites accumulation in the body and inhibition of bone marrow function. During acute exacerbations, almost all children with OAMD and AAMD displayed hyperammonemia. In contrast, patients with AAMD were more likely to exhibit glucose metabolic abnormalities. Among early-onset IEMs, OAMD had the highest number of cases associated with treatment abandonment, and death, accounting for approximately 63.3% (19/30). Following closely were AAMD, which accounted for about 36.6% (11/30) of cases, whereas children with early-onset FAOD had a relatively better prognosis. Although most IEMs may not have distinct clinical manifestations, most exhibit abnormalities in blood amino acids, acylcarnitines, and/or urinary organic acids. Therefore, the integration of LC-MS/MS and GC-MS/MS screening is a more efficient diagnostic strategy for suspected IEM cases.

However, there were several potential limitations in our study. Firstly, it was based on a single-center design, which may introduce population bias since the patient population within a single center might not fully represent the characteristics of the broader population. Secondly, not conducting genetic testing on all patients, especially a dropout rate of 12.66% (2,765/21,840), could have led to an underestimation of the incidence rate of IEMs and variant prevalence. And employing WES as the genetic testing method in this study, may also contribute to the underestimation due to its inherent limitations in identifying variants in non-coding regions, and specialized forms of genetic variations such as dynamic mutations, imprinting genes, and rearrangements. Thirdly, the lack of long term follow-up data on children with IEMs may also be a weakness of this study. Additionally, the interpretation of the identified genetic variants remains incomplete. Further functional studies will be necessary to elucidate the significance of these variants, particularly novel ones, and verify their association with specific IEMs. Future research will focus on conducting long term follow-up studies on neonates with IEMs, exploring potential environmental factors contributing to IEMs, investigating prenatal diagnosis methods for IEMs, and assessing the cost-effectiveness of comprehensive newborn screening programs for IEMs.

In summary, our study involved the screening of 21,840 hospitalized newborns using LC-MS/MS, GC-MS/MS and genetic analysis, leading to the confirmation of 58 cases of IEMs. The incidence of IEMs among hospitalized newborns was 1/370, with a higher incidence in full-term infants (1/420) than in premature infants (1/3,120). Among the identified IEMs, MMA, especially the isolated type, was the most prevalent, while NICCD and OTCD were more commonly observed in premature infants with IEMs. The combination of LC-MS/MS and GC-MS/MS was more efficient for screening IEMs, especially in cases with specific abnormal metabolites in the urine. Genetic testing was performed as soon as possible in the cases who metabolites markers in the blood and/or urine were atypical. Furthermore, our analysis identified 72 potentially variants in the 58 IEM cases, with 31.9% (23/72) of these sites being previously unreported. Our data contribute to the enrichment of the clinical features and genetic information related to IEMs and provide reliable laboratory evidence for future genetic counseling for the affected families.

## Data Availability

The original contributions presented in the study are included in the article/Supplementary Material, further inquires can be directed to the corresponding author.
