# Corrigendum: Disease spectrum, prevalence, genetic characteristics of inborn errors of metabolism in 21,840 hospitalized infants in Chongqing, China, 2017–2022

**DOI:** 10.3389/fgene.2024.1449534

**Published:** 2024-07-10

**Authors:** Dongjuan Wang, Juan Zhang, Rui Yang, Dayong Zhang, Ming Wang, Chaowen Yu, Jingli Yang, Wenxia Huang, Shan Liu, Shi Tang, Xiaoyan He

**Affiliations:** ^1^ Center for Clinical Molecular Medicine, National Clinical Research Center for Child Health and Disorders, Ministry of Education Key Laboratory of Child Development and Disorders, China International Science and Technology Cooperation Base of Child Development and Critical Disorders, Chongqing Key Laboratory of Pediatrics, Children’s Hospital of Chongqing Medical University, Chongqing, China; ^2^ Department of Neonatology, National Clinical Research Center for Child Health and Disorders, Ministry of Education Key Laboratory of Child Development and Disorders, Chongqing Key Laboratory of Pediatrics, Children’s Hospital of Chongqing Medical University, Chongqing, China

**Keywords:** inborn errors of metabolism, newborn screening, disease spectrum, genetic characteristics, tandem mass spectrometry

In the published article, there were four errors in Table 3 as published.

(1) For BCKDHA gene variant c.990_993delCTAC, the “ACMG” classification was erroneously listed as “US,” and it should be corrected to “LP”; the “Evidence” for the variant was incorrectly stated as “PM2_Supporting,” and it should be updated to “PVS1, PM2_Supporting.”
(2) For CPS1 gene variant c.381+1delG, the “ACMG” classification was erroneously listed as “US,” and it should be corrected to “LP”; the “Evidence” for the variant was incorrectly stated as “PM2_Supporting,” and it should be updated to “PVS1, PM2_Supporting.”
(3) For OTC gene variant chrX:g.34148019_38664751del, the “Evidence” was incorrectly stated as “1A(0) + 2A(1) + 3A(0) + 4L(0.45) = 1.45,” and it should be updated to “1A(0) + 2A(1) + 3A(0) = 1.”
(4) For PCCB gene variant c.898dupC, the “ACMG” classification was erroneously listed as “US,” and it should be corrected to “P”; the “Evidence” for the variant was incorrectly stated as “PM3_Strong, PM2_Supporting,” and it should be updated to “PVS1, PM3_Strong, PM2_Supporting.”

**TABLE 3:** The 23 previously unreported variants of 11 genes in patients with IEMs in this study.

IEM	Gene	Chr	Position	cDNA	Protein	Exon	ACMG	Evidence
MSUD	BCKDHA	19	41928907	c.1000G>A	p.G334R	8	LP	PM2_Supporting, PM5, PP3_Strong
		19	41928180	c.758C>T	p.A253V	6	US	PM2_Supporting, PM5, PP3_Moderate
		19	41928668	c.990_993delCTAC	p.T331Gfs*38	7	LP	PVS1, PM2_Supporting
	BCKDHB	6	80881035	c.670C>G	p.L224V	6	US	PM2_Supporting, PP3_Moderate
NICCD	SLC25A13	7	95818659–95818660	c.879delT	p.P293Lfs*61	9	LP	PVS1, PM2_Supporting
		7	95813572	c.1177+17C>G	Splicing	11	US	BP4
		7	95750528	c.2006C>G	p.S669*	18	US	PVS1_Moderate, PM2_Supporting
OTCD	OTC	X	38229066	c.234A>G	p.Q78Q	3	US	PM2_Supporting
		X	38280318	c.1048C>T	p.Q350*	10	US	PVS1_Moderate, PM2_Supporting
		X	34148019–38664751	chrX:g.34148019_38664751del	NA	NA	P	1A(0) + 2A(1) + 3A(0) = 1
CPS1D	CPS1	2	211441214	c.381+1delG	Splicing	3	LP	PVS1, PM2_Supporting
		2	211466972	c.1754T>C	p.M585T	16	US	PM2_Supporting, PP3_Strong
MMA	MMUT	6	49409658	c.1703C>A	p.A568D	10	US	PM2_Supporting, PP3_Strong
		6	49407987	c.1888G>A	p.G630R	11	LP	PM2_Supporting, PM5, PP3_Strong
	ACSF3	16	89167717	c.628A>C	p.K210Q	3	US	PP3_Strong
		16	89180853	c.1084A>T	p.M362L	6	US	PM2_Supporting
PA	PCCB	3	136016865	c.898dupC	p.L300Pfs*11	8	P	PVS1, PM3_Strong, PM2_Supporting
		3	135969398	c.181C>T	p.R61X	1	LP	PVS1, PM2_Supporting
		3	136016794	c.764G>T	p.G255V	8	US	PM2_Supporting, PP3_Strong
	PCCA	13	100953793	c.1145T>C	p.L382P	13	US	PM2_Supporting, PP3_Strong
MCCD	MCCC1	3	182759370	c.1252A>C	p.T418P	11	US	PM2_Supporting, PP3_Strong
		3	182751780–182751781	c.1679_1680insA	p.N560Kfs*10	14	P	PVS1, PM2_Supporting, PM3_Strong
VLCAD	ACADVL	17	7127300	c.1346A>C	p.E449A	14	US	PM2_Supporting, PP3_Strong

IEM, inborn errors of metabolism; Chr, Chromosome; MSUD, maple syrup urine disease; NICCD, neonatal intrahepatic cholestasis caused by citrin deficiency; OTCD, ornithine transcarbamylase deficiency; CPS1D, carbamyl phosphate synthase I deficiency; MMA, methylmalonic acidemia; PA, propionic acidemia; MCCD, 3-methylcrotonyl-coenzyme A carboxylase deficiency; VLCADD, very long-chain acyl-coenzyme A dehydrogenase deficiency; NA, no data available; P, pathogenic; LP, likely pathogenic; US, uncertain significance.

The correct Table 3 and its caption appear below.

In the published article, there were two errors. The text in the **Results and Discussion** sections referencing Table 3 requires updates to align with the corrected information of Table 3.

1. A correction has been made to **Results** Section, *Gene detection in patients with inherited metabolic disorders*, Paragraph 3.

The sentences previously stated:

“The pathogenicity of the 23 previously unreported variants mentioned above was analyzed using the ACMG rating system. Two mutations [a large 4.52 Mb hemizygous deletion containing the OTC gene (seq[hg19]del(X)(p21.1p11.4)chrX:g.34148019_38664751del) and c.1679_c.1680insA in the MCCC1 gene] were identified as pathogenic and 4 mutations (c.1000G>A in the BCKDHA gene, c.879delT in the SLC25A13 gene, c.1888G>A in the MMUT gene, and c.181C>T in the PCCB gene) were identified as likely pathogenic. The remainder were of uncertain significance, of which four were reported with different nucleotide changes at the same position, three variants were indexed in the Clinvar database but lacked relevant literature, and the remaining ten unreported variants of unknown significance included two frame-shift mutations, two termination mutations, two shear mutations and one intronic variant. The data were shown in Table 3.”

The corrected sentence appears below:

“The pathogenicity of the 23 previously unreported variants mentioned above was analyzed using the ACMG rating system. Three mutations [a large 4.52 Mb hemizygous deletion containing the OTC gene (seq[hg19]del(X)(p21.1p11.4)chrX:g.34148019_38664751del), c.898dupC in the PCCB gene and c.1679_c.1680insA in the MCCC1 gene] were identified as pathogenic and 6 mutations (c.1000G>A and c.990_993del CTAC in the BCKDHA gene, c.879delT in the SLC25A13 gene, c.381+1delG in the CPS1 gene, c.1888G>A in the MMUT gene, and c.181C>T in the PCCB gene) were identified as likely pathogenic. The remainder were of uncertain significance, of which four were reported with different amino acid substitutions at the same position, three variants were indexed in the Clinvar database lacked relevant literature, and the remaining seven unreported variants of unknown significance included a synonymous mutation, two termination mutations, three missense mutations and one intronic variant. The data were shown in Table 3.”

2. A correction has been made to **Discussion** Section, Paragraph 9.

The sentences previously stated:

“In addition, we analyzed 23 previously unreported genetic variants and evaluated their pathogenicity using the ACMG rating system. Among these, 2 variants were classified as pathogenic: a large 4.52 Mb hemizygous deletion containing the OTC gene (seq[hg19]del(X)(p21.1p11.4)chrX:g.34148019_38664751del) and the c.1679_c.1680 insA mutation in the MCCC1 gene. Furthermore, 4 variants were rated as likely pathogenic: c.1000G>A in the BCKDHA gene, c.879delT in the SLC25A13 gene, c.1888G>A in the MMUT gene, and c.181C>T in the PCCB gene. The remaining 17 unreported variants were categorized as uncertain significance by the ACMG system.”

The corrected sentence appears below:

“In addition, we analyzed 23 previously unreported genetic variants and evaluated their pathogenicity using the ACMG rating system. Among these, 3 variants were classified as pathogenic: a large 4.52 Mb hemizygous deletion containing the OTC gene (seq[hg19]del(X) (p21.1p11.4)chrX:g.34148019_38664751del), c.898dupC in the PCCB gene and the c.1679_c.1680insA mutation in the MCCC1 gene. Furthermore, 6 variants were rated as likely pathogenic: c.1000G>A and c.990_993delCTAC in the BCKDHA gene, c.879delT in the SLC25A13 gene, c.381+1delG in the CPS1D, c.1888G>A in the MMUT gene, and c.181C>T in the PCCB gene. The remaining 14 unreported variants were categorized as uncertain significance by the ACMG system.”

The authors apologize for these errors and state that these do not change the scientific conclusions of the article in any way. The original article has been updated.

