# Supplementary material for: Disease spectrum, prevalence, genetic characteristics of inborn errors of metabolism in 21,840 hospitalized infants in Chongqing, China, 2017-2022
Source: Front Genet. 2024 May 28;15:1395988. doi: 10.3389/fgene.2024.1395988 (PMC11165094; doi:10.3389/fgene.2024.1395988)
Supplement: Supplementary file 1 [file Table5.DOCX]

**Appendix table S2 The indicators and reference range of LC-MS/MS screening.**

| **No.** | **Indicators** | **Abbreviations** | **Reference range (μmol/L)** | **No.** | **Ratios** | **Reference range** |
| --- | --- | --- | --- | --- | --- | --- |
| 1 | Alanine | ALA | 100-800 | 43 | ARG/ORN | 0.15-0.4 |
| 2 | Arginine | ARG | 1-45 | 44 | CIT/ARG | 0.12-12 |
| 3 | Citruline | CIT | 6.5-40 | 45 | CIT/PHE | 0.12-0.7 |
| 4 | Glycine | GLY | 150-1100 | 46 | GLY/PHE | 2-15 |
| 5 | Leucine | LEU | 60-300 | 47 | LEU/PHE | 1.5-5.3 |
| 6 | Methionine | MET | 6-40 | 48 | MET/CIT | 0.5-3.5 |
| 7 | Ornithine | ORN | 40-350 | 49 | MET/PHE | 0.12-0.8 |
| 8 | Phenylalanine | PHE | 20-100 | 50 | ORN/CIT | 3-25 |
| 9 | Proline | PRO | 90-550 | 51 | PHE/TYR | 0.2-1.1 |
| 10 | Tyrosine | TYR | 30-350 | 52 | PHE/LEU | 0.1-0.53 |
| 11 | Valine | VAL | 50-300 | 53 | PRO/PHE | 1-10 |
| 12 | Free carnitine | C0 | 10.5-55 | 54 | VAL/PHE | 1-5 |
| 13 | Acetylcarnitine | C2 | 34-40 | 55 | C0/(C16+C18) | 2.5-50 |
| 14 | Propionylcarnitine | C3 | 0-4 | 56 | C3/MET | 0-0.3 |
| 15 | Malonylcarnitine+3-hydroxy butyrylcarnitine | C3DC+C4OH | 0-0.3 | 57 | C3/C0 | 0-0.18 |
| 16 | Butyrylcarnitine | C4 | 0-0.45 | 58 | C3/C2 | 0-0.22 |
| 17 | Methylmalonylcarnine+ 3-hydroxy isovalerylcarnitine | C4DC+C5OH | 0-0.4 | 59 | C3/C16 | 0.2-2.9 |
| 18 | Isovalerylcarnitine | C5 | 0-0.4 | 60 | (C3DC+C4OH)/C4 | 0.1-1.3 |
| 19 | Tiglylcarnitine | C5:1 | 0-0.02 | 61 | (C3DC+C4OH)/C8 | 0.5-5 |
| 20 | Glutarylcarnitine+3-hydroxy hexanoylcarnitine | C5DC+C6OH | 0-0.25 | 62 | (C3DC+C4OH)/C10 | 0.4-3.5 |
| 21 | Hexanoylcarnitine | C6 | 0-0.1 | 63 | (C3DC+C4OH)/C16 | 0-0.12 |
| 22 | Adipylcarnitine | C6DC | 0-0.18 | 64 | C4/C2 | 0-0.04 |
| 23 | Octanoylcarnitine | C8 | 0-0.15 | 65 | C4/C3 | 0-0.4 |
| 24 | Octenoylcarnitine | C8:1 | 0-0.35 | 66 | C4/C8 | 1.2-12 |
| 25 | Decanoylcarnitine | C10 | 0-0.22 | 67 | (C4DC+C5OH)/C3 | 0-0.4 |
| 26 | Decanoylcarnitine | C10:1 | 0-0.12 | 68 | (C4DC+C5OH)/C8 | 1-10 |
| 27 | Decadienoylcarnitine | C10:2 | 0-0.05 | 69 | C5/C0 | 0-0.02 |
| 28 | Dodecanoylcarnitine | C12 | 0-0.25 | 70 | C5/C2 | 0-0.04 |
| 29 | Dodecanoylcarnitine | C12:1 | 0-0.2 | 71 | C5/C3 | 0-0.35 |
| 30 | Myristoylcarnitine | C14 | 0-0.4 | 72 | (C5DC+C6OH)/C0 | 0-0.01 |
| 31 | Myrisoleylcarnitine | C14:1 | 0-0.25 | 73 | (C5DC+C6OH)/C3 | 0-0.18 |
| 32 | Tetradecadienoylcarnitine | C14:2 | 0-0.04 | 74 | (C5DC+C6OH)/(C3DC+C4OH) | 0.4-2.8 |
| 33 | 3-hydroxy myristoylcarnitine | C14OH | 0-0.03 | 75 | (C5DC+C6OH)/(C4DC+C5OH) | 0.1-1.2 |
| 34 | Palmitoylcarnitine | C16 | 0.5-6.5 | 76 | (C5DC+C6OH)/C8 | 0.5-4.8 |
| 35 | Hexadecenoylcarnitine | C16:1 | 0-0.4 | 77 | C8/C2 | 0-0.01 |
| 36 | 3-hydroxy palmitoleylcarnitine | C16:1OH | 0-0.07 | 78 | C8/C10 | 0.3-1.4 |
| 37 | 3-hydroxy palmitoylcarnitine | C16OH | 0-0.05 | 79 | C14:1/C2 | 0-0.013 |
| 38 | Octadecanoylcarnitine | C18 | 0.1-1.7 | 80 | C14:1/C12:1 | 1-5 |
| 39 | Octadecenoylcarnitine | C18:1 | 0.4-2.9 | 81 | C14:1/C16 | 0-0.1 |
| 40 | 3-hydroxy octadecenoylcarnitine | C18:1OH | 0-0.05 | 82 | (C16+C18:1)/C2 | 0.1-0.6 |
| 41 | Linoleylcarnitine | C18:2 | 0-0.6 | 83 | C16OH/C14 | 0-0.25 |
| 42 | 3-hydroxy octadecenoylcarnitine | C18OH | 0.01-0.03 | 84 | C16OH/C16 | 0-0.03 |
|  |  |  |  | 85 | C18OH/C18 | 0-0.4 |
