# Supplementary material for: Disease spectrum, prevalence, genetic characteristics of inborn errors of metabolism in 21,840 hospitalized infants in Chongqing, China, 2017-2022
Source: Front Genet. 2024 May 28;15:1395988. doi: 10.3389/fgene.2024.1395988 (PMC11165094; doi:10.3389/fgene.2024.1395988)
Supplement: Supplementary file 2 [file Table4.DOCX]

**Appendix Table S1 Baseline characteristics of the 21, 840 hospitalized infants in this study**

|  | **n** | **Constituent ratio, %** |
| --- | --- | --- |
| **Total** | **21,840** | **100.00** |
| **Gender** |  |  |
| Boy | 12,479 | 57.14 |
| Girl | 9,357 | 42.84 |
| Unknown gender | 4 | 0.02 |
| **Birth weight** |  |  |
| ≥ 4000 g | 729 | 3.34 |
| 2500 g ≤ birth weight < 4000 g | 13,529 | 62.94 |
| < 2500 g | 7,582 | 34.72 |
| **Gestational week** |  |  |
| Full term (≥ 37 weeks) | 12,874 | 58.95 |
| Premature (< 37 weeks) | 8,966 | 41.05 |
