# Supplementary material for: Disease spectrum, prevalence, genetic characteristics of inborn errors of metabolism in 21,840 hospitalized infants in Chongqing, China, 2017-2022
Source: Front Genet. 2024 May 28;15:1395988. doi: 10.3389/fgene.2024.1395988 (PMC11165094; doi:10.3389/fgene.2024.1395988)
Supplement: Supplementary file 3 [file Table6.DOCX]

**Appendix table S3 The indicators and reference range of GC-MS/MS screening.**

| **Indicators** | **Reference**  **range** | **Indicators** | **Reference**  **range** | **Indicators** | **Reference**  **range** | **Indicators** | **Reference**  **range** |
| --- | --- | --- | --- | --- | --- | --- | --- |
| Lactic-2 | 0-4.7 | 3-Methylglutaconic-2 | 0-0 | Isobutyrylglycine-1 | 0-6.4 | 2-Ketoglutaric-OX-2(2) | 0.3-21.3 |
| Aconitic-3 | 15.1-86.1 | 3-Methylglutaric-2 | 0-4.5 | 2-Keto-isovaleric-OX-2 | 0-0.1 | 3-Methylglutaconic-2 | 0-0 |
| Hexanoic-1 | 0-0 | Propionylglycine-2 | 0-0 | 2-OH-3-methylvaleric-2 | 0-0 | 2-Methyl-3-OH-butyric-1-2 | 0-0.3 |
| Glyoxylic-2 | 0-2.2 | Isobutyrylglycine-2 | 0-0 | Glutaric-2 | 0-4 | Succinylacetone-OX-2(1) | 0-0 |
| Oxalic-2 | 0-0 | Butyrylglycine-1 | 0-0 | 2-Keto-isocaproic-OX-2 | 0-0 | Vanilmandelic-3(VMA) | 11.7-84.6 |
| 3-OH-adipic-3 | 0.1-3.6 | 3-Methylglutaconic-2 | 0-4.2 | 2-Keto-adipic-OX-3 | 0-6.5 | 2-Propyl-3-OH-pentanoic(VPA)-2 | 0-0 |
| Glyoxylic-OX-2 | 0-6.1 | 3-OH-isovaleric-2 | 0-2.3 | 3-OH-propionic-2 | 0-1.1 | Mesaconic(Methylfumaric)-2 | 0-8.9 |
| Malic-3 | 0-0.7 | Decadienedionic-2 | 0-2.3 | 5-OH-methyl-2-furoic-1 | 0-0 | 2-Propyl-3-ketopentanoic(VPA)-2 | 0-0 |
| Pyruvic-OX-2 | 0-24.1 | 2-OH-butyric-2 | 0-0 | Methylcitric-4(2) | 0-1 | 2-Propyl-5-OH-pentanoic(VPA)-2 | 0-1.6 |
| Tiglyglycine-2 | 0-0 | 3-Methylglutaconic-2 | 0-2.9 | 3-OH--octenedioic-3 | 0-5.3 | 5-Oxoproline-2(pyroglutamic) | 0-7.6 |
| Tiglyglycine-1 | 0-0 | Isovalerylglycine-1 | 0-0.4 | 3-OH-suberic-3 | 0-4.8 | 2-Propyl-hydroxyglutaric(VPA)-2 | 0-0 |
| Octenedioic-2 | 0-0 | Butyrylglycine-2 | 0-0.7 | 2-OH-hippuric-3 | 0-0 | 6-Methylcrotonylglycine-1 | 0-0 |
| Suberic-2 | 0.3-4.7 | Methylsuccinic-2 | 0-6.4 | Indole-3-acetic-2 | 0-78.7 | 3-Methylcrotonylglycine-2 | 0-0 |
| Glyceric-3 | 0-1.6 | Methylmalonic-2 | 0.2-3.3 | Suberylglycine-2 | 0-0 | Citric-4 | 31.4-572.3 |
| Malonic-2 | 0-0.1 | Isovalerylglycine-2 | 0-0 | Acetoacetic-OX-2 | 0-0 | Hippuric-1 | 6.2-284.1 |
| Glutaconic-2 | 0-0 | 2-Hexenedioic-2 | 0-16.4 | 2-OH-sebacic-3 | 0-5.3 | 2-Keto-3-methylvaleric-OX-2 | 0-0 |
| Uracil-2 | 0-7 | 3-methyladipic | 0-23.3 | 3-OH-sebacic-3 | 0-4.4 | 2-Methyl-3-OH-valeric-2(1) | 0-0 |
| Adipic-2 | 0-5 | Thiodiglycolic-2 | 0-0 | 2-OH-hippuric-2 | 0-17.6 | 2-Methyl-3-OH-valeric-2(2) | 0-0 |
| Sebacic-2 | 0.4-7 | 7-OH-octanoic-2 | 0-0 | Dodecanedioic-2 | 0-0 | 3-(3-OH-phenyl)-3-OH-propionic-3 | 0-0 |
| Urea-2 | 104.6-763 | Valproic(VPA)-1 | 0-0 | N-Acetyltyrosine-3 | 0-0 | 4-OH-phenyllactic(PHPLA)-2 | 0-7 |
| Pimelic-2 | 0-9.3 | 3-OH-butyric-2 | 0-3.7 | 2-OH-isobutyric-2 | 0-0 | 4-OH-phenylpyruvic(PHPPA)-OX-2 | 0-0.9 |
| Orotic-3 | 0-1.5 | 2-OH-glutaric-3 | 0.6-5.9 | 3,6-Epoxydodecanedioic-2 | 0-5.2 | 3-OH-isobutyric-2 | 0-9 |
| Palmitic-1 | 0-13.8 | 3-OH-glutaric-3 | 0-0 | 3-OH-dodecanedioic-3 | 0-1.4 | 3-OH-valeric-2 | 0-0 |
| Acetoacetic | 0-0 | Phenyllactic-2 | 0-4.9 | 3,6-Epoxyteradecanedioic-2 | 0-3.9 | Mevalonolactone-2 | 0-0 |
| Fumaric-2 | 0-7.3 | 4-OH-butyric-2 | 0-0 | 3-OH-3-methylglutaric-3 | 0-25.7 | Mevalonolactone-1 | 0-0 |
| Benzoic-1 | 0-18.7 | 4-OH-benzoic-2 | 0-7.8 | 3-OH-phenylacetic-2 | 0-0.9 | Acetylglycinr-1 | 0-0.1 |
| Decanoic-1 | 0-0.4 | Homogentisic-3 | 0-1.4 | Uric-4 | 0-7.2 | Propionylglycine-1 | 0-0 |
| Octanoic-1 | 0-0.4 | Hexanoylglycine-1 | 0-0 | Azelaic-2 | 0-10.7 | 2-OH-isocaproic-2 | 0-0 |
| Vanillic-2 | 0-0 | Phenylpyruvic-OX-2 | 0-0 | Ethylmalonic-2 | 0-6.2 | 2-OH-isovaleric-2 | 0-0 |
| 2-OH-adipic-3 | 0-2 | N-Acetylaspartic-2 | 0-3.7 | Hippuric-2 | 0-11.7 | 4-OH-phenylacetic | 8.6-73.2 |
| Glycerol-3 | 0-0.8 | Acetylglycine-1 | 0-0 | Isocitric-4 | 8.3-29 | Ethylhydracrylic-2 | 0-2.9 |
| Phosphoric-3 | 0-43 | Methylcitric-4(1) | 0-11 | Phenylacetic-1 | 0-0.4 | Maleic-2 | 0-0.4 |
| Succinic-2 | 6.5-65.8 | 2-Deoxytetronic | 0-6.3 | Homovanillic-2(HVA) | 5.8-24.9 | 2-Ketoglutaric-OX-2(1) | 3-102.9 |
